# Evaluating the Mental Health of Physician-Trainees Using an SMS Text Message–Based Assessment Tool: Longitudinal Pilot Study

**DOI:** 10.2196/45102

**Published:** 2023-06-02

**Authors:** Nicole H Goldhaber, Annie Chea, Eric B Hekler, Wenjia Zhou, Byron Fergerson

**Affiliations:** 1 Department of Surgery School of Medicine University of California, San Diego Health La Jolla, CA United States; 2 Herbert Wertheim School of Public Health University of California, San Diego La Jolla, CA United States; 3 MEMOTEXT Corporation Toronto, ON Canada; 4 Department of Anesthesiology School of Medicine University of California, San Diego Health La Jolla, CA United States

**Keywords:** physician burnout, mental health, text-message assessment, text, mobile text, stress assessment, text message, pilot study, physician, burnout, United States, survey, trainee, stress, engagement, users, tracking

## Abstract

**Background:**

Physician burnout is a multibillion-dollar issue in the United States. Despite its prevalence, burnout is difficult to accurately measure. Institutions generally rely on periodic surveys that are subject to recall bias. SMS text message–based surveys or assessments have been used in health care and have the advantage of easy accessibility and high response rates.

**Objective:**

In this pilot project, we evaluated the utility of and participant engagement with a simple, longitudinal, and SMS text message–based mental health assessment system for physician-trainees at the study institution. The goal of the SMS text message–based assessment system was to track stress, burnout, empathy, engagement, and work satisfaction levels faced by users in their normal working conditions.

**Methods:**

Three SMS text message-based questions per week for 5 weeks were sent to each participant. All data received were deidentified. Additionally, each participant had a deidentified personal web page to follow their scores as well as the aggregated scores of all participants over time. A 13-question optional survey was sent at the conclusion of the study to evaluate the usability of the platform. Descriptive statistics were performed.

**Results:**

In all, 81 participants were recruited and answered at least six (mean 14; median 14; range 6-16) questions for a total of 1113 responses. Overall, 10 (17%) out of 59 participants responded “Yes” to having experienced a traumatic experience during the study period. Only 3 participants ever answered being “Not at all satisfied” with their job. The highest number of responses indicating that participants were stressed or burnt out came on day 25 in the 34-day study period. There were mixed levels of concern for the privacy of responses. No substantial correlations were noted between responses and having experienced a traumatic experience during the study period. Furthermore, 12 participants responded to the optional feedback survey, and all either agreed or strongly agreed that the SMS text message–based assessment system was easy to use and the number of texts received was reasonable. None of the 12 respondents indicated that using the SMS text message–based assessment system caused stress.

**Conclusions:**

Responses demonstrated that SMS text message–based mental health assessments are potentially useful for recording physician-trainee mental health levels in real time with minimal burden, but further study of SMS text message–based mental health assessments should address limitations such as improving response rates and clarifying participants’ sense of privacy when using the SMS text message–based assessment system. The findings of this pilot study can inform the development of institution-wide tools for assessing physician burnout and protecting physicians from occupational stress.

## Introduction

Physician burnout is an enormous problem across the nation; it was present prior to the COVID-19 pandemic but was also amplified by the pandemic [1]. Defined as “a syndrome of exhaustion, cynicism, and decreased effectiveness at work,” physician burnout affects the quality and productivity of the health care system and the personal lives of physicians themselves [2]. According to a selection of recent reports on prepandemic physician burnout, the prevalence of physician burnout symptoms was greater than 40% [3-5]. With the rise of physician burnout and increased advocacy for physician well-being, studies have mostly retrospectively explored risk factors for this type of occupational stress and interventions that may potentially be used to address it—such as mindfulness, exercise, and building community [4].

When evaluating physician well-being, accurate and reliable measurements of physician burnout must be used. Currently, there are several retrospective secondary use–type methods of gathering existing data to measure physician burnout, including the use of existing data collected from electronic health record–usage logs, physician vacation-day usage, clinician turnover rates, and reports on clinical effort [6]. Alternative methods used to measure physician well-being include survey- or questionnaire-type measurement tools such as the Maslach Burnout Inventory (MBI) [7]. However, criticisms of the MBI include its length (22 items), the lack of utility for developing interventions, and risk for recall biases [8]. Alternatively, single-item measures have been found to be effective for measuring burnout in physicians and medical students [9].

SMS text message–based surveys have been utilized in health care and have the advantage of easy accessibility and high response rates [10-12]. In this pilot project, we trialed a simple, engaging, longitudinal, and single-item SMS text message–based survey system to evaluate physician-trainee mental health. We then evaluated the utility, usability, and burden of the SMS text message–based assessment system. Ultimately, the goal of these assessments was to monitor the stress levels faced at work by physicians at our institution.

## Methods

### Study Design

This study was a longitudinal pilot study at the University of California, San Diego Health Medical Center (UCSDH), to determine if an SMS text message–based assessment program can efficiently track physician mental health. A collaboration between a third-party company—MEMOTEXT—and UCSDH was formed to create the SMS text message platform.

### Sample Recruitment

All participants recruited were physicians in training (residents or fellows) working at UCSDH, which was the only inclusion criteria for this study. Participants were recruited through email and word of mouth during October to November 2021. There were no specific exclusion criteria for this study.

### Study Protocol

The enrollment survey collected the following participant data to provide basic demographic information and allow for further contact: phone number, email, and department. Three SMS text message–based questions per week for 5 weeks were sent to participants; they were asked to reply with a single number from the scale provided (between 1 and 5), for example, “On a scale of 1-5 how stressed are you right now?” (Additional examples can be found in Table 1). The question sent was randomly chosen. Weekdays and times (between 9 AM and 4 AM) were also randomly assigned. The method of randomization was done through MEMOTEXT’s platform using an SQL function. Participants were asked to answer each prompt based on how the participant felt at the moment of the reply. Replies were anonymous. One reminder SMS text message was sent if there was no reply received within 1 hour. On the Monday of the sixth week, each participant received 1 additional text question, “Have you endured a traumatic event in the last 5 weeks?” If the answer is yes to this question, this participant’s subsequent responses were analyzed in the context of the traumatic effect accordingly.

**Table 1:** SMS text message–based questions.

Question or statement	Response				
	1	2	3	4	5
How satisfied are you with your job?	Not at all satisfied	Not too satisfied	Neither satisfied nor dissatisfied	Somewhat satisfied	Very satisfied
To what extent does the following statement describe you: “I am an empathetic person.”	Very untrue for me	Untrue for me	Neither true nor untrue	True for me	Very true for me
Stress means a state in which a person feels tense, restless, nervous or anxious or is unable to sleep at night because his/her mind is troubled all the time. Do you feel this kind of stress?	Very much	Somewhat	Neither stressed nor unstressed	Not too much	Not at all
To what extent does the following statement describe you: “I feel burned out from my work.“	Very true for me	True for me	Neither true nor untrue	Untrue for me	Very untrue for me
Work engagement means a positive, fulfilling, work-related state of mind that is characterized by vigor, dedication, and absorption. Do you feel this kind of work engagement?	Not at all	Not too much	Neither engaged nor disengaged	Somewhat	Very much

### Ethics Approval

The study protocol was approved by the University of California, San Diego, Institutional Review Board (IRB# 803280). Participation was voluntary, and informed consent was obtained through the enrollment survey. All data collected for the study were deidentified for the research team. Participants were not compensated and could opt out at any time.

Participants were also given access to follow their scores, as well as the anonymous aggregated scores of all participants, over time in a web-based “Personal Dashboard.” A 13-question optional survey was sent to participants at the conclusion of the study to evaluate the usability of the platform. No additional direct incentives were provided for participants.

### Statistical Analysis

For data analysis, MEMOTEXT provided processed data (graphs of aggregated participant scores) on a weekly basis. Each participant was assigned a deidentified subject ID. The response (a number between 1-5), the time and date of response, the day and week of the study, participant’s department, and the type of question asked were recorded. The total number of questions each participant responded to was also recorded. In addition, Microsoft Excel was utilized for basic demographic descriptive statistics.

## Results

**Figure 1:**
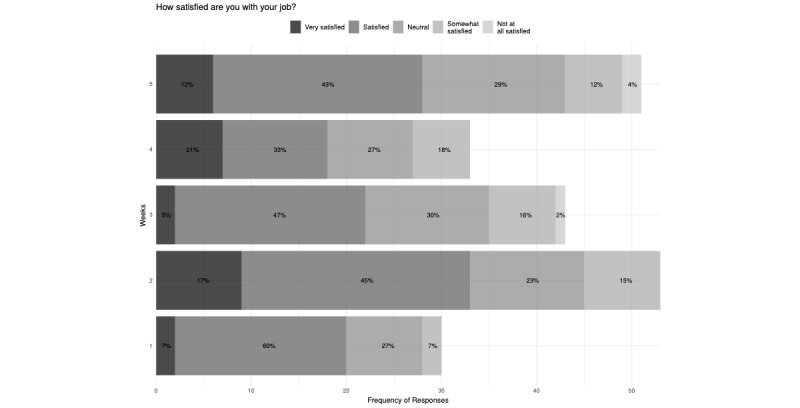
Participant response distribution to the job satisfaction question.

In all, 81 resident physicians and fellows at UCSDH were recruited to participate in this study, with representation from the following departments and divisions: anesthesia (n=34, 42.0%), family medicine (n=1, 1.2%), geriatrics (n=1, 1.2%), infectious disease (n=11, 13.6%), obstetrics and gynecology (n=17, 21.0%), psychiatry (n=1, 1.2%), pathology (n=1, 1.2%), and surgery (n=15, 18.5%). Participants answered at least six (mean 14; median 14; range 6-16) questions for a total of 1113 responses. Overall, 10 (17%) out of 59 participants responded “Yes” to having experienced a traumatic experience during the study period. Only 3 participants ever answered being “Not at all satisfied” with their job (during week 3: 1/43, 2% and week 5: 2/30, 7%; Figure 1).

**Figure 2:**
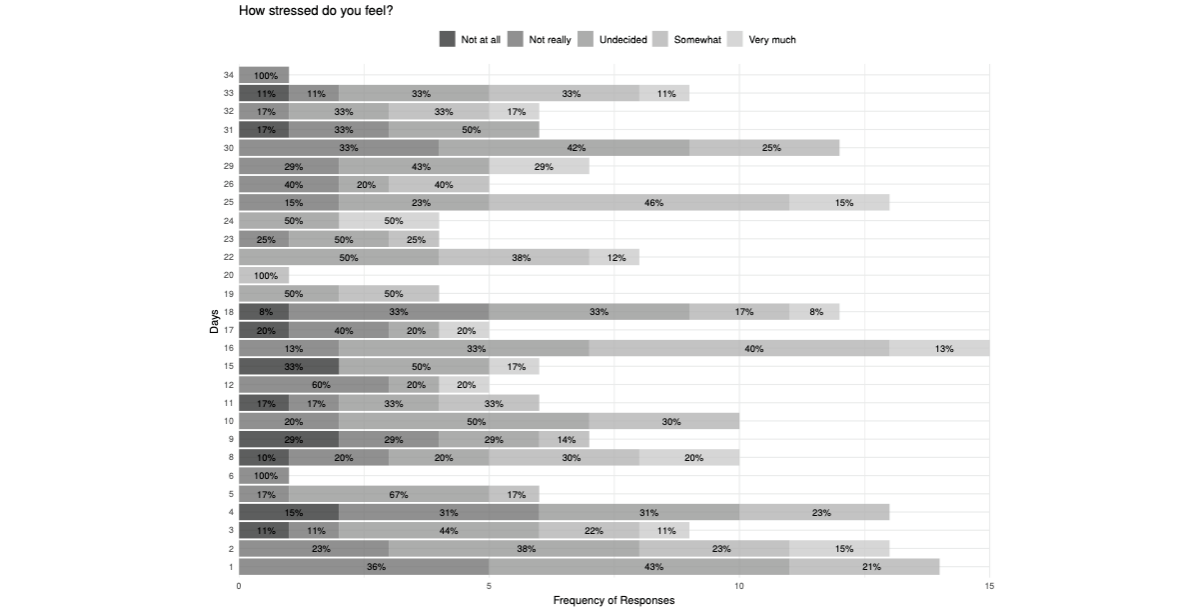
Participant response distribution to the stress assessment question across the study period.

**Figure 3:**
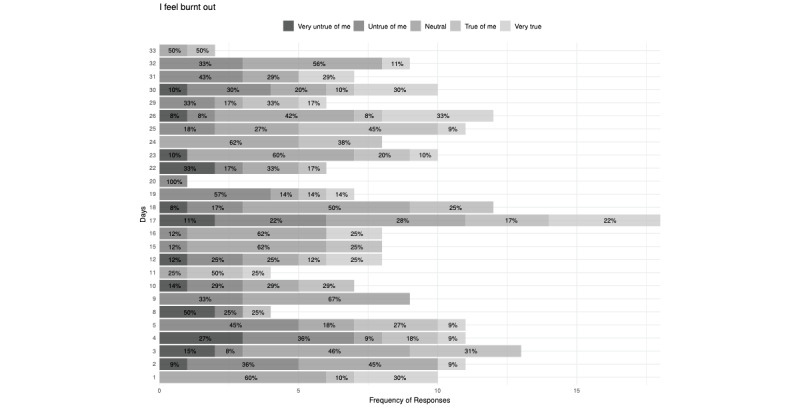
Participant response distribution to burnout assessment across the study period.

The highest number of responses indicating that the participants were stressed (“Somewhat (2)” or “Very much (1)”; 8/13, 61%) or burnt out (“True of me (2)” or “Very true (1)”; 6/11, 54%) came on day 25 in the 34-day study period (Figures 2 and 3). No substantial correlations were noted between responses and having experienced a traumatic experience during the study period.

In all, 12 participants responded to the optional feedback survey following the program. All respondents either “Agreed” (n=3, 25%) or “Strongly Agreed” (n=9, 75%) that the text assessment system was easy to use. All respondents either “Agreed” (n=6, 50%) or “Strongly Agreed” (n=6, 50%) that the number of texts received was reasonable. All respondents either “Strongly Disagreed” (n=3, 25%), “Disagreed” (n=8, 67%), or “Somewhat Disagreed” (n=1, 8%) that using the SMS text message–based stress assessment system caused stress. There was a mix of level of concern for privacy of responses: “Strongly Disagree” (n=2, 17%), “Disagree” (n=4, 33%), “Neither Agree nor Disagree” (n=3, 25%), “Agree” (n=2, 17%), and “Strongly Agree” (n=1, 8%).

## Discussion

This project demonstrates that the SMS text message–based stress assessment system is a tool that has potential for recording participant mental health in real time and over time, as shown by participants’ responses to questions regarding usability and burden. Overall, our findings are concordant with the limited literature on SMS text message–based stress assessments for physicians. One Swedish research team previously used weekly SMS text messages to monitor physician occupational stress and successfully measured individual levels of stress and linked them to objective workload measures [13]. To our knowledge, there have not been any further studies on the usability of SMS text message–based stress assessments for physicians nor physician-trainees.

In addition to being a promising tool for measuring physician well-being, SMS text message–based surveys can also serve as behavioral reinforcements and reminders of mindfulness. The implementation of SMS text messaging services acting as mental health interventions (daily supportive psychoeducational texts) has been shown to effectively reduce symptoms of stress [14,15]. Our study population included physician-trainees for this pilot phase and can be generalized to all physicians given that differences in burnout have been shown to be relatively minimal between physician-trainees and early career physicians [16]. Future studies could provide supportive well-being SMS text messages as an intervention to increase self-awareness and mindfulness for physicians specifically and utilize the same SMS text message as a method of surveying well-being to begin addressing the systematic problem of physician burnout.

Strengths of this study include the cost- and time-effective execution of data collection for both the participants and the researchers. Because collecting longitudinal data in the form of lengthy surveys from already busy physicians can present a substantial time burden and can be subject to recall bias, SMS text message–based assessments effectively minimize these potential weaknesses, as the texts are sent out in real time. Considering the frequency that an average person, including physicians, uses their phone, sending out SMS text message–based surveys may be the most accurate way for researchers to collect prospective data in real time. Previously mentioned retrospective methods of evaluating physician burnout, such as the MBI, can be subject to recall bias because those tools ask “in the last X days, have you experienced...” as opposed to immediate assessment and response to an SMS text message upon reading it [7]. The efficacy of SMS text message monitoring in a clinical setting has previously been demonstrated to be user-friendly and have high response rates [11].

Limitations of this pilot study include the small sample size from a single institution, low response rates in the final survey, mixed level of concern in privacy, and potential intraindividual variability of stress over time. Small-scale recruitment was done in only 1 institution and was skewed toward inpatient physician-trainees. The feedback survey at the end of this study protocol was optional, resulting in only 12 (14.8%) responses out of 81 total participants. Together with mixed levels of concern regarding the privacy of the SMS text message–based stress assessment system, this may indicate underreporting, especially for sensitive questions regarding work satisfaction, stress, burnout, and traumatic experiences. Future studies should aim for more robust recruitment and further reassurance of privacy to enforce a sense of psychological safety. Intraindividual variability of stress over time is a factor that is hard to control due to the randomization of the time that the SMS text messages were sent out during the week, as well as the experiences physicians may see on their day-to-day job, which the tool aims to assess.

The findings of this pilot study show promise and encourage the pursue of future research utilizing this tool on a larger scale. Future studies should include a larger sample size and higher response rates across multiple training levels, multiple departments, and ultimately multiple institutions. Furthermore, researchers could potentially include comparison groups for utilizing SMS text message–based mental health assessments for other occupational groups (eg, nurses and non–health care occupations with high occupational stress). As more literature is published on this topic, future studies can utilize different study designs, such as cohort study or randomized controlled trials, including stress intervention methods to shift data toward a more casual nature. The ultimate goal of these studies would be to inform the development of institution-wide intervention efforts to protect physicians from occupational stress and prevent physician burnout at a systematic level.

## Abbreviations

MBI: Maslach Burnout Inventory
UCSDH: University of California, San Diego Health Medical Center

## Data Availability

The data sets generated during and/or analyzed during this study are available from the corresponding author on reasonable request.
